# Key barriers to the provision and utilization of maternal health services in low-and lower-middle-income countries; a scoping review

**DOI:** 10.1186/s12905-024-03177-x

**Published:** 2024-06-05

**Authors:** Yaser Sarikhani, Seyede Maryam Najibi, Zahra Razavi

**Affiliations:** 1 Research Center for Social Determinants of Health, Jahrom University of Medical Sciences, Jahrom, Iran; 2 Research Center for Traditional Medicine and History of Medicine, Department of Persian Medicine, School of Medicine, Shiraz University of Medical Sciences, Shiraz, Iran

**Keywords:** Maternal health, Maternal Health Services, Access, Utilization, Provision, Barriers

## Abstract

**Background:**

The preservation and promotion of maternal health (MH) emerge as vital global health objectives. Despite the considerable emphasis on MH, there are still serious challenges to equitable access to MH services in many countries. This review aimed to determine key barriers to the provision and utilization of MH services in low- and lower-middle-income countries (LLMICs).

**Methods:**

In this scoping review, we comprehensively searched four online databases from January 2000 to September 2022. In this study, the approach proposed by Arksey and O’Malley was used to perform the review. Consequently, 117 studies were selected for final analysis. To determine eligibility, three criteria of scoping reviews (population, concept, and context) were assessed alongside the fulfillment of the STROBE and CASP checklist criteria. To synthesize and analyze the extracted data we used the qualitative content analysis method.

**Results:**

The main challenges in the utilization of MH services in LLMICs are explained under four main themes including, knowledge barriers, barriers related to beliefs, attitudes and preferences, access barriers, and barriers related to family structure and power. Furthermore, the main barriers to the provision of MH services in these countries have been categorized into three main themes including, resource, equipment, and capital constraints, human resource barriers, and process defects in the provision of services.

**Conclusions:**

The evidence from this study suggests that many of the barriers to the provision and utilization of MH services in LLMICs are interrelated. Therefore, in the first step, it is necessary to prioritize these factors by determining their relative importance according to the specific conditions of each country. Consequently, comprehensive policies should be developed using system modeling approaches.

**Supplementary Information:**

The online version contains supplementary material available at 10.1186/s12905-024-03177-x.

## Background

Maternal care encompasses a series of interventions aimed at mitigating the effects of risk factors, managing illnesses, and ultimately safeguarding the well-being of both women and children. Maternal health (MH) services are concerned with maintaining the health of women before and during pregnancy, during childbirth, and in the postnatal period. Maternal care, which involves a broad spectrum of services including screening, early disease detection, prompt treatment, and health education, plays a vital role in decreasing mortality rates and improving women’s health outcomes [1]. Despite the advancements in medical science and the provision of guidelines and operational instructions, health policymakers have consistently prioritized the maintenance and improvement of MH. This concern is especially prominent in low-income countries, where addressing the issue remains a top priority [2]. The significance of maternal mortality extends beyond being a mere indicator of poor health conditions; it also represents a formidable challenge for healthcare systems [3].

Annually, over 500,000 women across the globe lose their lives due to pregnancy and childbirth-related complications. It is noteworthy that developing countries experience an alarming 99% of maternal deaths, underscoring the pressing need for targeted interventions to address this issue [4]. Despite the particular focus on MH, the maternal mortality rate in 2019 was 145 per 100,000 live births worldwide. Meanwhile, in developing countries, this ratio is estimated at 276 deaths for every 100,000 live births [5]. Studies also show that maternal mortality decreased from 11.2 to 5.01 per 100,000 population worldwide between 1999 and 2019. In low-income countries, this index fell from 43.31 to 21.10. Moreover, from 1999 to 2019, the rate of disability-adjusted life years (DALYs) in the 100,000 population due to maternal disorders decreased from 695 to 394 globally and from 2,536 to 1,262 in low-income countries [6]. Consequently, even though the global maternal mortality rates are decreasing, there remains a substantial disparity between the average global rates and those observed in low-income countries. This emphasizes the critical need to prioritize MH services in numerous nations.

According to available reports, the main direct factors associated with maternal death and injury are heavy bleeding, infections, hypertension, and unsafe abortion, while the main indirect causes are anemia, malaria, and heart disease [4]. Meanwhile, the goal of maternal care standards is to improve access to effective services, make the efficient use of available resources to achieve desired outcomes, help healthcare providers improve the quality of services, improve people’s satisfaction, and promote the use of services [7]. Nonetheless, even with the considerable attention given to maternal care, numerous obstacles hinder the successful implementation of maternal care programs. These challenges are present at both the level of mothers as recipients of services and the level of service providers. Numerous research from different parts of the world have investigated the barriers to accessing MH care services. In 2020, Shibata et al. showed that prenatal care utilization was significantly associated with geographic location, household income, and education level [8]. Transportation to access health facilities [9–11], high cost of services [12, 13], and lack of competence of health professionals [10–13] were also among the barriers mentioned in different studies.

Effective MH care is vital for reaching the health-related Sustainable Development Goals (SDGs). In this context, adopting comprehensive strategies is essential for the provision of suitable MH services and the reduction of maternal mortality rates globally [14]. Identifying key obstacles to the provision and utilization of MH services can provide policymakers with insights to develop the necessary strategies to address and overcome these obstacles. In light of this matter, it is feasible to provide communities with timely and high-quality services to address the pressing challenges of reducing maternal mortality. This is especially vital for low-income countries [15], as it can substantially contribute to the improvement of public health. Therefore, this study aims to determine the main barriers to the provision and utilization of MH services in low- and lower-middle-income countries (LLMICs), using a scoping review approach.

## Methods

We used a scoping review method to identify barriers to the provision and utilization of MH services. A comprehensive review was conducted, resulting in the creation of an evidence map associated with the topic. The provision of mental health services is largely determined by the socioeconomic conditions of communities. Therefore, this study investigated these factors in LLMICs to leverage the findings for policy interventions in countries with similar contexts. The classification of countries is based on the information provided by the World Bank. Countries with a gross national income (GNI) per capita of less than US$1,045 were classified as low-income countries using the Atlas method. Additionally, countries where the above index ranged from $1046 to $4095 were classified as lower-middle-income countries [16].

A scoping review is used in this study because this type of review provides the possibility of involving studies with different designs and sampling methods [17]. This type of research also allows for the identification of key components of a topic to provide a map of evidence and reveal the research gap in the considered area [18]. In this study, the approach proposed by Arksey and O’Malley was used to perform a scoping review. This approach involves five separate steps: 1- determining the research question, 2- finding and extracting studies, 3- selecting relevant studies, 4- tabulating data, and 5- summarizing information, analyzing themes, and presenting results [17].

### Determining the research question

The scope and extent of a review study is usually determined by the research question. However, because scoping research has a continuous and iterative process of searching, selecting articles, and modifying the research question, the research question of this review study was finalized during the study process. In this study, barriers to the provision and utilization of MH services in LLMICs were considered as the expected result. This research was conducted to answer the question: “What are the main barriers to the provision and utilization of MH services in LLMICs?”

### Finding and extracting studies

Before conducting a comprehensive search, the research team searched the Cochrane database and other databases to ensure that there were no comparable reviews. Subsequently, four main databases were searched systematically for articles published between January 2000 and September 2022. These databases are PubMed, Scopus, Web of Science, and ScienceDirect. The Google Scholar search engine and scientific society websites were also searched for reports and other publications. To retrieve related articles, relevant words were searched in three fields, including title, abstract, and keywords. According to the initial review of similar studies and to perform a more precise search, the keywords have been divided into 2 groups and according to the methodology of the scoping review, these groups were modified and completed during the study. The keywords and phrases in each group were combined with the logical operator “OR”, and the search results from each group were combined with other groups with the logical operator “AND”. The search keywords were determined by reviewing the keywords indexed in the MeSH and Emtree databases, as well as the corresponding entry terms associated with these databases. Table 1 shows the search strategy of the review. In this study, Endnote 20 software was used to manage references.

**Table 1:** The search strategy of the study

Searching Databases	PubMed, Scopus, Web of Science, ScienceDirect, Google Scholar
Limitations	Language: Articles with full text in English Time: Articles published from 1/1/2000
Search Strategy	**#1 AND #2**
**#1**	“Maternal care” OR “Maternal care services” OR “Maternal health services” OR “Prenatal care” OR “Preconception care” OR “Postnatal care” OR” Perinatal care”
**#2**	Delivery OR Provision OR Providing OR Utilization OR Use OR Usage OR Access OR Accessibility OR Availability

### Selecting relevant studies

To select articles related to the review objective, the research team conducted a three-step iterative assessment process. In this procedure, the three stages involved are scanning the title, abstract, and full text, respectively. At each step, the search strategy was modified, and new articles were searched for and assessed.

In all three phases, the assessment was performed in parallel and independently by two members of the research team. To become more familiar with the different steps of the study, two researchers conducted a preliminary pilot study. we used three criteria of scoping reviews to develop the research question, as well as at all steps of the assessment. Accordingly, mothers, barriers to the provision and utilization of MH services, and LLMICs were considered as “population, concept, and context” (PCC), respectively. Within the scope of this study, healthcare provision is characterized as the process of providing health interventions through the integration of resources such as funds, personnel, facilities, and medications to meet health requirements. Moreover, healthcare utilization pertains to the degree to which individuals make use of healthcare services, which is influenced by their level of awareness, the availability, and accessibility of these services, as well as their satisfaction with the quality of care provided. Finally, the concept of maternal care encompasses the provision of services and support to women before and during pregnancy, at the time of childbirth, and in the postpartum period, with the primary objective of ensuring the overall well-being of both the mother and the child.

To improve the validity of the results, the quality of the selected articles was assessed using standard checklists. To do this, we used two tools, including the strengthening the reporting of observational studies in epidemiology (STROBE) checklist [19] and the critical appraisal skills program (CASP) checklists [20]. At all stages of the evaluation, cases of disagreement were reviewed by the third researcher, and the final decision was made.

Due to limitations related to the translation of the texts, only articles with the full text in English were selected. Moreover, given that changes in socio-economic status impact the utilization and provision of MH services, articles published since 2000 were selected for analysis to examine the most recent research in this field. We only included original studies and debate articles, so review articles and articles published in the form of letters to the editor and commentaries were not included in the analysis.

### Tabulating data

To extract data from the selected articles, we created a data charting form. For each article retrieved, this form contains information, including the author’s name, article title, year of publication, publishing journal, research design, and the main results of the article. To this end, the researchers participated in the previous phases, jointly extracted the data and continuously filled in the data charting form. The data charting form is provided as Additional file 1.

### Summarizing information, analyzing themes, and presenting results

To synthesize and analyze the extracted data, we used the method proposed for this purpose, namely qualitative content analysis [21]. Two researchers independently analyzed the data and then compared the results so that cases of agreement were determined and confirmed and cases of disagreement were identified and resolved. In the first phase of the thematic analysis, two researchers became familiar with the data by reading the texts several times and then extracted the primary codes according to the research objectives. In the next step, the researchers interpreted the primary codes and determined the categories, sub-themes, and main themes. Sub-themes and main themes were then evaluated and reviewed by two researchers. In the final phase, the research team held a joint meeting, where the themes were revised and if necessary, they were combined, separated, or deleted, and finally, the themes were named according to their conceptual context. The results of the qualitative themes analysis are presented in the form of a table. Tables related to thematic analysis are provided in Additional file 2. To allow a better understanding of the extent of the evidence, the table of themes also includes information on the number of articles used to develop each theme. Finally, the thematic network resulting from the analysis was designed as a conceptual framework of evidence to provide a more comprehensive insight into the topic.

To ensure the trustworthiness and rigor of the results, the criteria proposed by Guba and Lincoln were used, including credibility, confirmability, dependability, and transferability. To enhance the credibility of the findings, we have benefited from long-term engagement with the texts and the use of peer checks in the analyses. To improve confirmability, we asked two experts in the field of qualitative research to confirm the accuracy of the data analysis process. To achieve dependability, the study process was explained clearly and in detail. In this regard, four external reviewers assessed the study protocol. Finally, to enhance transferability, comprehensive details of the study process are provided to allow replication by other similar studies [22].

## Results

**Fig. 1:**
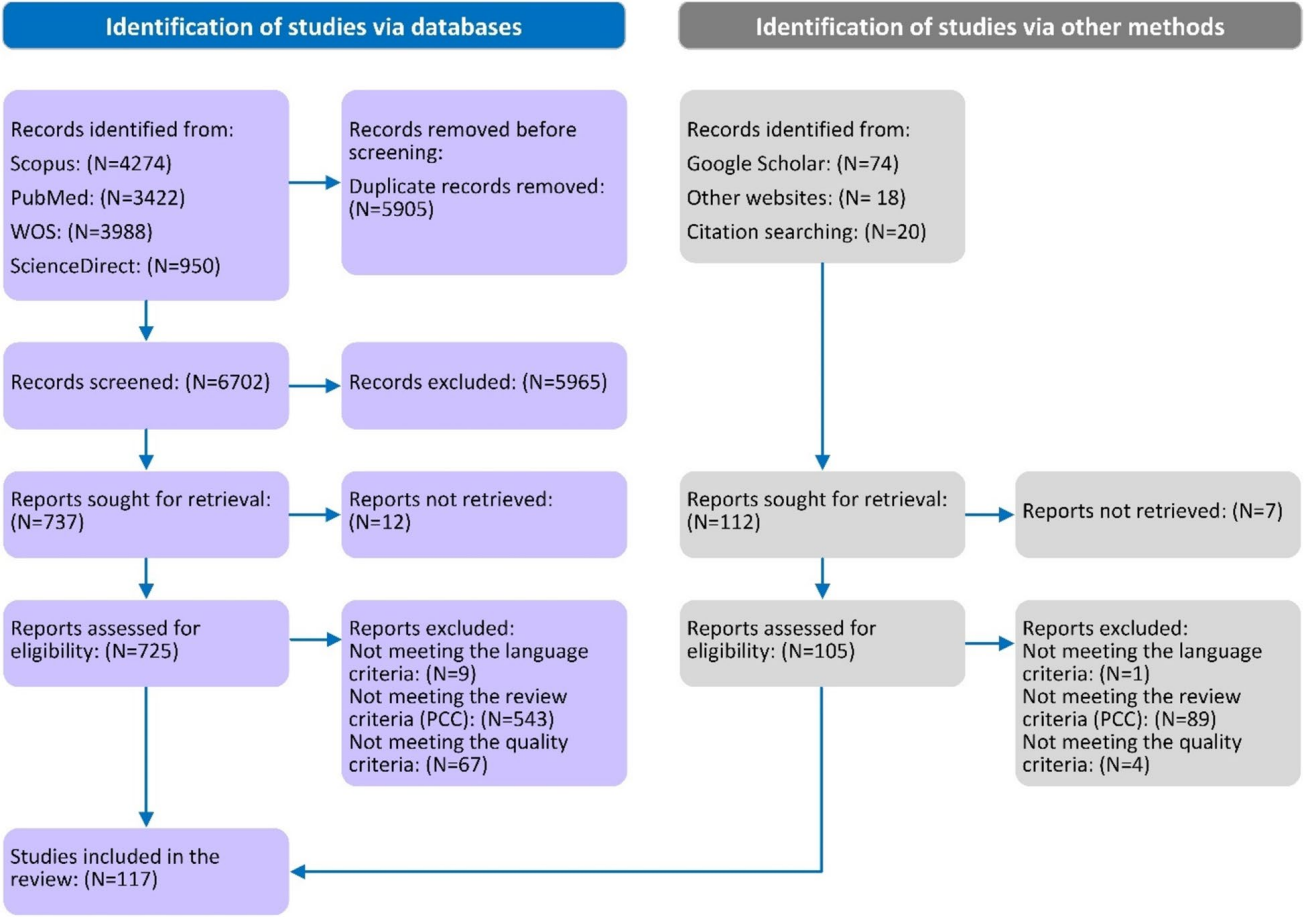
PRISMA flowchart of the study

Our search in various databases resulted in the extraction of 12,719 articles. After removing the duplicates, 6814 articles entered the analysis phase. In the first review phase, we scanned the titles and 849 articles entered the second review stage. In the second phase, the article abstracts were reviewed and the items that did not match the scope of the study were removed. Based on this, we selected 293 articles for further analysis. In the third step, the full text of the remaining articles was assessed and 117 articles were selected for final analysis [23–139]. In Fig. 1, the article selection process is outlined through a PRISMA flowchart. Table 2 presents the characteristics of the articles used in the thematic analysis. Of the selected articles, 57 (49%) focused on utilization challenges, 17 (14%) on provision barriers, and 43 (36%) explored both types of barriers. The highest number of articles published per year was from 2016 to 2022 with an average of more than 12 articles per year. More than 66% (*N* = 78) of the studies were conducted in Africa, with Ethiopia and Nigeria having the highest number of articles with 27 (23%) and 16 (13.6%) studies, respectively. Among Asian countries, India had the most articles (*N* = 6, 5.1%).

**Table 2:** Characteristics of the articles selected for the final analysis

Characteristics	Frequency and (%) of included articles
Publication year	
2001–2005	3 (2.5%)
2006–2010	10 (8.5%)
2011–2015	19 (16.2%)
2016–2020	61 (52.1%)
2021≤	24 (20.5%)
Study Location	
Africa	78 (66.6%)
Asia	32 (27.3%)
America and Oceania	4 (3.4%)
Multi-country	3 (2.5%)
Service	
Maternal care	54 (46.1%)
Perinatal care	9 (7.8%)
Postnatal care	29 (24.7%)
Preconception care	11 (9.4%)
Prenatal care	14 (12.0%)
Study type	
Quantitative	70 (59.8%)
Qualitative	38 (32.4%)
Mixed methods	9 (7.8%)

**Fig. 2:**
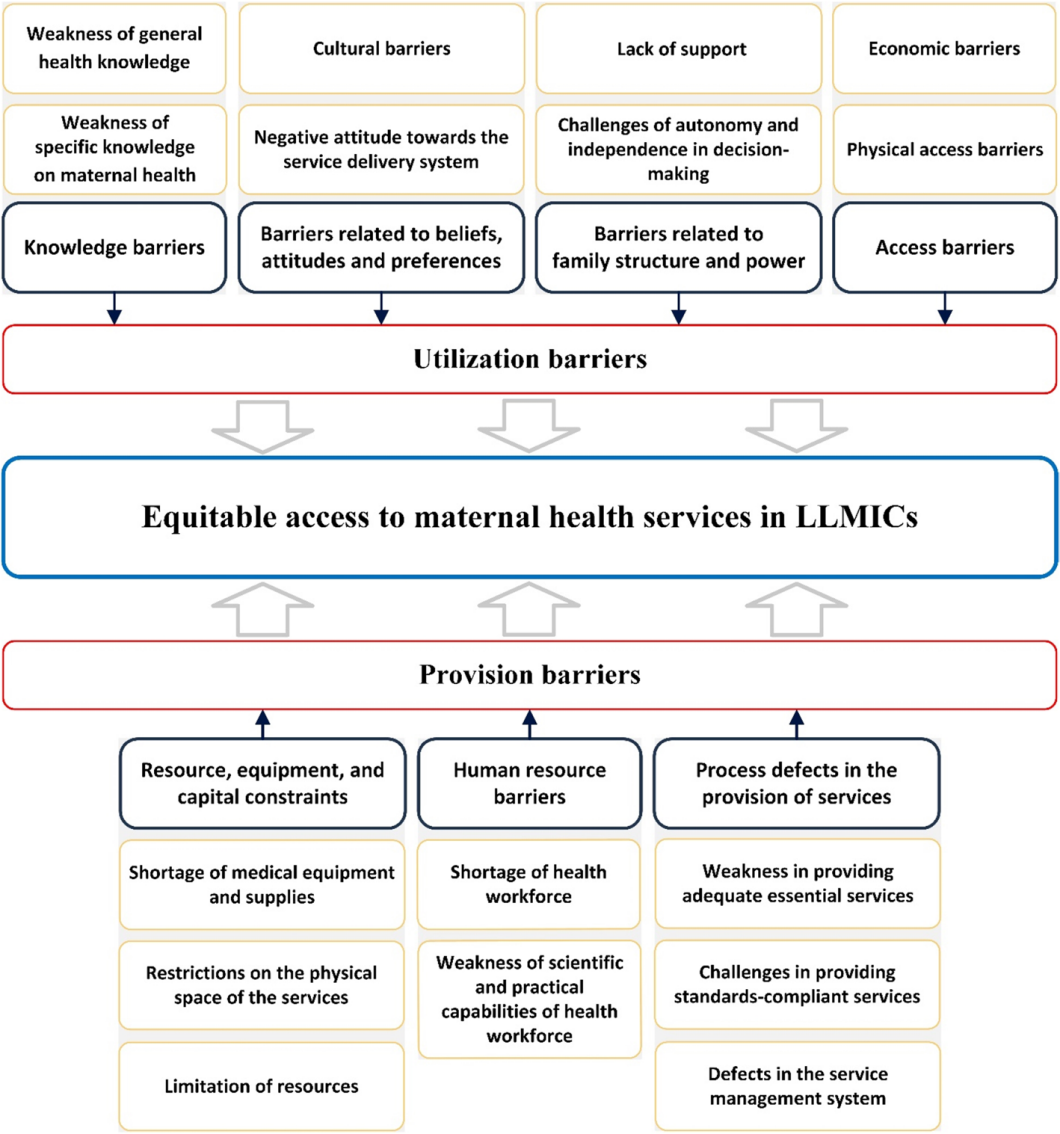
Conceptual framework of evidence on key barriers to the utilization and provision of MH services in LLMICs

The thematic analysis led to the identification of four main themes and eight sub-themes regarding the barriers to the utilization of MH services, the results of which are presented in Table 3. Additionally, three main themes and eight sub-themes related to challenges in the provision of MH service were identified, and presented in Table 4. The conceptual framework of evidence resulting from thematic analysis is presented in Fig. 2.

**Table 3:** Themes and sub-themes related to the challenges of using MH services

Themes	Sub-themes	Included Studies	
		Frequency (%)	References
Knowledge barriers	Weakness of specific knowledge on maternal health	56 (47.86)	[23–29, 31–33, 36–42, 47–49, 53, 55, 56, 61, 63, 66, 68–71, 73, 76–78, 81, 83, 85, 87, 89, 90, 92, 96, 99, 100, 103, 104, 106, 109, 112, 120, 122, 128, 129, 133, 134, 138]
	Weakness of general health knowledge	16 (13.67)	[33, 37, 48, 55, 84, 88, 94, 95, 97, 100, 106, 113, 116, 124, 131, 134]
Barriers related to beliefs, attitudes, and preferences	Negative attitude towards the service delivery system	11 (9.40)	[26, 31, 37, 42, 49, 78, 81, 87, 91, 104, 129]
	Cultural barriers	28 (23.93)	[25, 28, 31, 36, 38, 42, 48, 49, 52, 55, 62, 72–74, 76, 81, 82, 92, 100, 104, 108, 111, 115, 123–125, 128, 129]
Access barriers	Economic barriers	13 (11.11)	[24, 30, 45, 46, 63, 66, 73, 80, 88, 94, 102, 117, 137]
	Physical access barriers	23 (19.65)	[25, 28, 34, 46, 49, 63, 64, 66, 74, 76, 81, 88, 95, 100, 101, 104, 105, 115, 118, 124, 126, 134, 138]
Barriers related to family structure and power	Challenges of autonomy and independence in decision-making	32 (27.35)	[24, 30–32, 34, 41, 45, 46, 48, 50, 52, 53, 55, 59, 60, 62, 71, 74, 79, 81, 88, 92, 100, 104, 107, 109, 112, 115, 120, 121, 123, 139]
	Lack of support	10 (8.54)	[25, 28, 31, 44, 63, 67, 70, 76, 88, 110]

**Table 4:** Themes and sub-themes related to the challenges of MH service provision

Themes	Sub-themes	Included Studies	
		Frequency (%)	References
Resource, equipment, and capital constraints	Shortage of medical equipment and supplies	16 [13, 67]	[31, 35, 39, 42, 46, 63, 66, 76, 81, 86, 98, 104, 114, 123, 132, 135]
	Restrictions on the physical space of the services	14 [11, 96]	[31, 35, 63, 72, 75, 76, 93, 104, 111, 120, 127, 129, 132, 135]
	Limitation of resources	4 (3.41)	[25, 73, 93, 113]
Human resource barriers	Shortage of health workforce	22 (18.80)	[31, 35, 39, 42, 46, 50, 58, 63, 66, 72–76, 86, 98, 100, 114, 123, 129, 134, 135]
	Weakness of scientific and practical capabilities of the health workforce	26 (22.22)	[35, 36, 42, 43, 58, 63, 65, 66, 75, 76, 81, 86, 96, 100, 103, 104, 110, 113, 115, 118–120, 123, 127, 129, 135]
Process defects in the provision of services	Challenges in providing standards-compliant services	11 (9.40)	[35, 42, 49, 50, 66, 74, 76, 103, 120, 127, 135]
	Defects in the service management system	9 (7.69)	[58, 63, 67, 76, 81, 98, 103, 113, 135]
	Weakness in providing adequate essential services	31 [26, 49]	[28, 30, 37, 41, 47, 48, 54, 57, 63, 66, 68, 72–76, 95, 100, 103, 108, 109, 112, 118, 119, 127, 129, 130, 133, 134, 136]

### A) barriers to the utilization of MH services

#### A-1- knowledge barriers

This theme explains the barriers to the utilization of MH services related to the weakness of general and specific health knowledge among women.

##### Weakness of specific knowledge on MH

The results of this review showed that women’s poor knowledge of MH is the most frequent barrier (N: 56, 47.86%) to the utilization of MH services in LLMICs. The low level of specific knowledge on MH is reflected in three areas, including a lack of awareness of risk factors and danger signs [89, 90, 106], failure to receive special care and information in previous stages of care [26, 87, 138] and lack of awareness of available health services [27, 55, 122].

##### Weakness of general health knowledge

The results showed that the lack of general health knowledge among women is a major barrier to the use of MH services. Lack of awareness of health needs [100, 113] and limited access to media and information [55, 84, 124] are two key challenges in this area.

#### A-2- barriers related to beliefs, attitudes, and preferences

This theme represents the barriers to the use of MH services related to perceptual and cultural aspects of women in LLMICs.

##### Negative attitude towards the service delivery system

The results show that women’s negative attitudes towards the health system in LLMICs can be seen as a barrier to the optimal use of MH services. This challenge can be explained in three categories including, negative attitudes toward services [78, 129], negative attitudes towards the competence of service providers [31, 49], and negative experiences from past services [81, 91].

##### Cultural barriers

The findings of the review showed that cultural aspects can be powerful barriers to the use of MH services. The results of the thematic analysis led to the explanation of five challenges in this area, namely Self-treatment tendencies [92, 111], cultural and traditional customs barriers [36, 74], preferences for using traditional services [108, 128], sense of shame and fear [81, 82], and communication barriers [31, 92].

#### A-3- Access barriers

This theme represents the barriers that limit the accessibility of MH services. The main challenges explained in this theme were the financial and physical barriers.

##### Economic barriers

The results of the current study revealed that poor insurance coverage [30, 73] and financial restrictions [63, 66] are two major economic challenges that hinder the use of maternal care services in LLMICs.

##### Physical access barrier

This challenge explains the limitations of the transportation system [95, 100, 118]. This problem is particularly significant for the residents of remote and rural areas.

#### A-4- barriers related to family structure and power

This theme expresses the barriers that prevent women from accessing MH services due to discriminatory power structures within the family. The challenges of autonomy and decision-making as well as support issues are explained in this theme.

##### Challenges of autonomy and independence in decision-making

The results indicate that this challenge was the second most frequent problem in the studies analyzed. The results of the thematic analysis showed that limited autonomy and low decision-making power [31, 32] as well as dependence on spouses in decision-making [88, 100] are two main barriers related to women’s decision-making power that prevent them from using MH services in LLMICs.

##### Lack of support

This review revealed that a lack of support from relatives can be a barrier to the utilization of MH services. This challenge can be divided into two categories including, lack of support from family members [44, 67] and lack of spousal support [70].

### B) barriers to the provision of MH services

#### B-1- resource, equipment, and capital constraints

This theme explains the barriers to the provision of MH services related to the lack of medical equipment and medicines, financial resources, and physical capital.

##### Shortage of medical equipment and supplies

 The results of this review showed that in more than 13% of studies conducted in LLMICs, the problem of lack of equipment [98, 114] and medicines [66, 104] was mentioned as the challenge of providing MH services.

##### **Restrictions on the physical space of the services**

This challenge explains the barriers that limit service due to physical space issues. in this context, three categories were determined, namely limitations of service delivery space [76, 93], insufficient infrastructure [24, 75], and inappropriate service delivery environment [35, 72].

##### **Limitation of resources**

The results of the study indicated that the limited financial resources [58, 100] and shortage of physical capital [93] are two barriers to the provision of optimal MH services in LLMICs.

#### B-2- human resource barriers

This theme explains the problems in the provision of MH services caused by the shortage of health human resources and the low competency and efficiency of health professionals.

##### **Shortage of health workforce**

The results of this study showed that the shortage of qualified health workers [42, 66] and the consequent limitation of working hours [86, 100] are among the most important obstacles to the provision of MH services in LLMICs.

##### **Weakness of scientific and practical capabilities of the health workforce**

The findings of this study indicated that the weakness of scientific and practical capabilities of the health workforce was the second most frequent challenge in the provision of MH services. This challenge can be divided into four categories including, the negative attitude of the health workforce [66, 96], incompetence of health professionals [58, 103], inadequate knowledge of service providers [35, 113], and Insufficient motivation of service providers [58, 76].

#### B-3- process defects in the provision of services

This theme explains barriers to the provision of MH services that arise due to defects in management processes, non-compliance with standard policies, and inability to deliver necessary services.

##### **Challenges in providing standards-compliant services**

The results of this review indicated that the shortage of appropriate guidelines [42, 103] and the low quality of services [49, 127] are two important challenges in the provision of MH services in LLMICs.

##### **Defects in the service management system**

This review revealed that lack of integrity in the service provision system [113, 135], weakness of managerial processes [67, 76], poor management of the information system [58], and political restrictions [58] are four categories of this challenge.

##### **Weakness in providing adequate essential services**

The findings of this review indicated that the weakness in providing adequate essential services was the barrier most frequently mentioned in the studies carried out in LLMICs (N:31, 26,49%). This challenge was divided into two categories including, lack of availability of necessary services [73, 127, 129, 134] and long waiting list [118, 123].

## Discussion

Preserving the health of mothers and children is a fundamental objective in global health agendas [140] given that insufficient access to MH care can contribute to elevated maternal mortality rates [141]. The SDGs highlight the importance of achieving equitable access to maternal and child health care services globally. Despite the setting of goals and action plans, 94% of maternal deaths still take place in low-and middle-income countries, which are often preventable [142]. Research on the determinants that impact mothers’ health reveals that MH is a social construct with various effects that are influenced by contextual factors [143]. This highlights the need for comprehensive evidence to ensure the provision of effective services. Accordingly, this study aimed to explain the challenges associated with providing and utilizing MH services in LLMICs. Numerous studies have emphasized the impact of some demographic factors, such as education and income, on the use of MH services. The final analysis of this study did not include demographic characteristics, as the focus was on elucidating the fundamental and systemic obstacles linked to the underutilization of MH services. The analysis resulted in the identification of four main themes and eight sub-themes regarding the barriers to the utilization of MH services. Moreover, three main themes and eight sub-themes regarding challenges in the provision of MH service were identified. The research findings are discussed in this section, categorized by each theme and sub-theme.

### Barriers to the utilization of MH services

The findings of this review indicate that the most frequent barrier to the utilization of MH services in LLMICs is the knowledge barrier. Earlier studies have shown that low levels of education in low-income countries have an impact on women’s awareness of the advantages of MH care and can occasionally discourage them from using services even when those services are easily accessible [144, 145]. This is because being able to utilize a service and having knowledge of its existence does not always indicate that a woman has a thorough understanding of its purpose [120]. Furthermore, the continued use of MH care is an important issue related to women’s level of knowledge and education [146, 147], so that educated women have better health-seeking behavior and more knowledge about MH services [148]. The issue of limited access to education and scarce opportunities for health information is particularly prevalent in rural communities. Women residing in these areas face significant challenges in obtaining knowledge about maternal and general health, which further exacerbates the problem [149]. In light of this matter, it is crucial to prioritize the empowerment of women through education and enhance their understanding of MH by employing culturally sensitive communication strategies [8]. The development of educational strategies should consider the contextual nuances of rural and urban communities, incorporate community-based health promotion programs, and follow the basic literacy levels of the residents [25]. It is also necessary to use the capacity of the media to keep MH services fully informed [146] because women exposed to the media have better knowledge and attitudes toward the danger signs of pregnancy and childbirth, which will lead to increased use of MH services [150].

Barriers related to family structure and power are among the other barriers to the utilization of MH services, which are mentioned in 35% of the studies included in this review. In this theme, the lack of independence and autonomy and the lack of support for mothers are issues that have been explained as barriers to the use of MH services. Decision-making about MH services and reproductive rights depends on the interaction between women, their partners, and other family members. Restricting these opportunities can hinder access to MH services [50]. In this regard, studies show that women’s independence in decision-making or joint decision-making by husband and wife increases the likelihood of using MH care [151, 152]. Although the concept of women’s autonomy is intricate and challenging to measure, there is no widely agreed-upon definition or method for its evaluation [153], previous studies show that women’s limitation in decision-making is related to several underlying factors, such as women’s educational attainment [50, 153], family socioeconomic status [50], age [153, 154], urbanity [154], monthly income [155, 156], and patriarchal culture in some societies [157, 158]. In a meta-analysis study, Natnael estimated decision autonomy for MH services in low- and middle-income countries at 55.16% [154]. Meanwhile, Tiruneh’s study found that, among many other factors, women’s decision-making power was the strongest predictor of increased use of MH services [159]. For this reason, the 2030 SDGs consider women’s empowerment and gender inequality as essential components of public health interventions [154]. Therefore, there should be more strategies to encourage men’s participation in MH-related activities through couple counseling to increase utilization of MH services [157]. It is also suggested to expand women’s access to education to improve their skills and self-confidence, which in turn increases women’s ability to make decisions about health care and personal development [154].

Barriers related to beliefs, attitudes, and preferences are among other barriers to the utilization of MH services, which include cultural barriers and challenges related to negative attitudes towards the service provision system. To overcome cultural barriers, community-based dialogue and consultation are needed to understand concerns about MH care utilization, as well as specific reasons for avoiding or delaying care [25]. In addition, measures such as cultural adaptation of maternity care, increasing men’s involvement in MH care, community mobilization and involvement, launching health education campaigns, and challenging social beliefs and practices that limit access and use of MH services, are also suggested in this area [160].

Economic and physical access barriers were among the other challenges in using MH services explained in our study. Direct and indirect costs of services are among the major barriers to MH care utilization, which have been mentioned repeatedly in the studies included in this review. In low-income countries, families with low socioeconomic status face high out-of-pocket expenses and indirect costs, such as transportation expenditures to access health services, which can lead to financial hardship [161]. Even though low-income countries experience a large share of the global burden of diseases, they allocate only a small part of their budget to the healthcare sector [162]. Consequently, governments in low-income countries must make adequate investments to eliminate impediments to both economic and physical access to healthcare services [9].

### Barriers to the provision of MH services

While social, economic, and cultural factors play a role in determining the utilization of MH services, it is essential to address barriers on the service provider side to enhance MH outcomes [163].

Among the studies analyzed in this review, process defects in the provision of services emerged as the most prevalent obstacle to the provision of MH services. This theme explains weaknesses in providing adequate essential services, challenges in providing standards-compliant services, and defects in the service management system. The lack of comprehensive and integrated services, a poor referral system, and the lack or poor implementation of guidelines are among the main barriers to the provision of MH care in LMICs, making it difficult to deliver high-quality services. Referral plays a crucial role in the healthcare system by facilitating the transfer of women to an appropriate healthcare facility, thereby ensuring the provision of quality services and reducing the risk of maternal mortality. This is even though some maternal mortality is attributed to non-standard care at the referral level [164]. An effective referral system requires adequate coordination and communication between the different levels of care. However, according to previous studies, the communication cycle between referral levels is often problematic in low-income countries due to the lack of an appropriate transport system [58, 98, 115, 165]. Consequently, due to the importance of the referral system in the development of quality and integrated services in low-income countries, the availability of a reliable transportation system plays an important role in the timely delivery of MH services and facilitates referrals between different levels of care [58].

This review led to the explanation of human resource barriers and resource, equipment, and capital constraints as other key challenges to MH service provision. Healthcare providers like physicians, midwives, and nurses play a crucial role in maternal and child health services, and numerous deficiencies within the healthcare system stem from the constraints of existing services and the scarcity of proficient health professionals [166]. Insufficient allocation of resources towards health human resources, medical equipment, and medicines, coupled with inadequate infrastructure including roads, electricity, and water, along with deficiencies in the referral system, will greatly impede the delivery of services in low-income countries [12]. On the other hand, health professionals in low-income countries work in challenging environments with limited systemic support, such as poor management and coordination of staff, lack of motivation due to low wages, and lack of infrastructure such as electricity or water supply. Additionally, the dearth of supervision and training opportunities can impact the quality of services and the performance of professionals [76, 167]. The development of training modules, the improvement of the payment system, and the consideration of non-financial incentives can be used as strategies to enhance the motivation of health professionals, resulting in increased staff performance and the quality of MH services. Moreover, the lack of modern medical equipment in some low-income countries reduces the effectiveness of maternal care providers. As a result, while promoting local investment, the governments of these nations need to collaborate with international organizations to secure funding for medical equipment and medicines, as well as to implement practical strategies to enhance sustainable supply chain management [168].

### Knowledge gap

The examination of available literature in this review has brought to light a significant trend, indicating that the majority of studies conducted during the specified time frame focused on identifying barriers to achieving equitable access to MH services in African nations. Conversely, a limited number of studies were dedicated to investigating these pertinent issues in Asian and Latin American countries. Consequently, due to the specific socioeconomic context of each region, it is essential for future research in these countries to meticulously investigate the associated challenges and solutions. Moreover, considering the scarce resources available in low-income countries, there is a dearth of thorough investigations examining the relative importance and weight of each factor that affects equitable access to MH services. Determining the weight and significance of these factors can assist in prioritizing them for interventions, thus enabling the provision of more precise evidence to inform related policies and actions. A meticulous evaluation of the findings of this study suggests a complex interdependence among many of the barriers to equitable access to MH services within a systemic structure. Therefore, in light of the importance of systems thinking, the World Health Organization has emphasized employing this approach to solve health system issues. Accordingly, it is necessary to conduct studies using system modeling to provide a holistic approach to all key factors influencing equitable access to MH services, taking into account their interrelationships, through the development of causal models, and thus provide optimal solutions.

### Limitations of the study

One significant limitation of this study was the exclusion of studies written in languages other than English, mainly because of translation challenges. Moreover, the diverse range of MH services and the publication of articles and reports with widely dispersed titles and keywords made it difficult to search, evaluate, and choose articles for this study. Accordingly, despite the extensive attention devoted to the review process, certain studies may have been omitted.

## Conclusions

Based on the findings of this review, the main challenges in the utilization of MH services in LLMICs are explained under four main themes including, knowledge barriers, barriers related to beliefs, attitudes and preferences, access barriers, and barriers related to family structure and power. Financial barriers, lack of support, cultural obstacles, and deficiencies in general and specialized knowledge are the main issues within this domain. Furthermore, the main barriers to the provision of MH services in these countries have been categorized into three main themes including, resource, equipment, and capital constraints, human resource barriers, and process defects in the provision of services. This area is confronted with several critical problems, including a problematic medicine and equipment supply chain, a weak management system, inadequate financial and physical resources, and an inefficient human resource management system. The conclusions drawn from this study reveal that research efforts in the subject of this review have been unevenly distributed among LLMICs, highlighting the potential for additional research in many nations. The evidence from this study suggests that many of the factors identified in this review are interrelated. Therefore, in the first step, it is necessary to prioritize these factors by determining their relative importance according to the specific conditions of each country. Consequently, comprehensive policies should be developed using system modeling approaches.

### Supplementary Information

Supplementary Material 1.

Supplementary Material 2.

## Data Availability

No datasets were generated or analysed during the current study.

## Abbreviations

CASP: Critical appraisal skills program
DALY: Disability-adjusted life year
GNI: Gross national income
LLMICs: Low-and lower-middle-income countries
MH: Maternal health
STROBE: Strengthening the reporting of observational studies in epidemiology
SDGs: Sustainable development goals
