# Supplementary material for: Key barriers to the provision and utilization of maternal health services in low-and lower-middle-income countries; a scoping review
Source: BMC Womens Health. 2024 Jun 5;24:325. doi: 10.1186/s12905-024-03177-x (PMC11151574; doi:10.1186/s12905-024-03177-x)
Supplement: Supplementary file 1 — Supplementary Material 1. [file 12905_2024_3177_MOESM1_ESM.docx]

**Data Carting Form**

| **NO** | **Author** | **Title** | **Journal** | **Country** | **Year (P)** | **Design** | **Service** | **Key Findings** |
| --- | --- | --- | --- | --- | --- | --- | --- | --- |
| 1 | Teshome Abuka Abebo | Postnatal care utilization and associated factors among women of reproductive age Group in Halaba Kulito Town, Southern Ethiopia | Archives of Public Health | Ethiopia | 2018 | Cross-sectional | Postnatal | **Utilization**:  1- Didn’t aware of at least one postpartum danger signs. |
| 2 | Abel Ntambue ML | Determinants of maternal health services utilization in urban settings of the Democratic Republic of Congo--a case study of Lubumbashi City | BMC Pregnancy and Childbirth | Congo | 2012 | Cross-sectional | Perinatal | **Utilization**:  1- Not used antenatal care. |
| 3 | Tafesse Lamaro Abota | Postnatal Care Utilization and Associated Factors among Married Women in Benchi-Maji Zone, Southwest Ethiopia: A Community Based Cross-Sectional Study | Ethiopian Journal of Health Sciences | Ethiopia | 2018 | Cross-sectional | Postnatal | **Utilization**:   1. Low Rate of antenatal care follow-up. 2. Low awareness about problems of postnatal period. |
| 4 | Stephen O. Abrokwah | The effect of social health insurance on prenatal care: the case of Ghana | Int J Health Care Finance Econ | Ghana | 2014 | Quantitative | Prenatal | **Utilization**:  1- Lack of health insurance coverage |
| 5 | Ramesh Adhikari | Effect of Women's autonomy on maternal health service utilization in Nepal: A cross sectional study | BMC Women's Health | Nepal | 2016 | Cross-sectional | Postnatal | **Utilization**:  1- Low level of autonomy |
| 6 | K E Agho | Population attributable risk estimates for factors associated with non-use of postnatal care services among women in Nigeria | BMJ | Nigeria | 2016 | Quantitative | Postnatal | **Utilization**:   1. Poor knowledge of delivery-related complications. 2. Limited or no access to the mass media. |
| 7 | Rolle Remi Ahuru | Non-utilization of Primary Healthcare Centres for Skilled Pregnancy Care among Women in Rural Communities in Delta State, Southern Nigeria: Perspectives from Mothers, Fathers, and Healthcare Providers | Journal of International Women's Studies | Nigeria | 2021 | Qualitative | Prenatal | **Utilization**:   1. Distance barrier and poor road network/lack of transportation. 2. Non-Affordability of Delivery Charges 3. Women were submissive to their husbands.   **Provision**:   1. Lack of medical equipment 2. Lack of availability of drugs 3. Centers were always locked, especially at night. |
| 8 | Nursena Aksunger | What prevents pregnant women from adhering to the continuum of maternal care? Evidence on interrelated mechanisms from a cohort study in Kenya | BMJ Open | Kenya | 2022 | Prospective cohort | Prenatal | **Utilization**:  1- Delayed antenatal care |
| 9 | M.K. Akter | The challenges of prenatal care for Bangladeshi women: a qualitative study | International Nursing Review | Bangladesh | 2018 | Qualitative | Prenatal | **Utilization**:   1. Women’s lack of opportunity to make decision; 2. Pregnancy as anormal life event; 3. Insufficient money for prenatal care; 4. Lack of family support; 5. No permission to go to hospital without a guardian; 6. Inconvenient transportation. |
| 10 | Nazmul Alam | The role of transportation to access maternal care services for women in rural Bangladesh and Burkina Faso: A mixed methods study | International Journal of Gynecology and Obstetrics | Multi-country | 2016 | Mixed methods | Perinatal | **Utilization**:  1- Lack of reliable transportation |
| 11 | Alenoghena IO | Maternal health services uptake and its determinants in public primary health care facilities in edo state, Nigeria | The Nigerian Postgraduate Medical Journal | Nigeria | 2015 | Mixed methods | Maternal | **Utilization**:   1. Self-assessment of health   **Provision**:   1. Non-clean environment |
| 12 | Pierre Kébreau Alexandre | Prenatal care utilization in rural areas and urban areas of Haiti | Rev Panam Salud Publica | Haiti | 2005 | Quantitative | Prenatal | **Utilization**:   1. Not aware where to go for pregnancy complications 2. Distance to nearest health center |
| 13 | Rana Ejaz Ali Khan | Quantity of Prenatal Care (PNC) services use in Southern Punjab: A case study of Bahawalpur | J Pak Med Assoc | Pakistan | 2019 | Quantitative | Prenatal | **Utilization**:   1. Woman's freedom of movement, 2. Having knowledge about pregnancy complications |
| 14 | Elias Amaje | Utilization of Preconception Care and Its Associated Factors among Pregnant Women of West Guji Zone, Oromia, Ethiopia, 2021: A Community-Based Cross-Sectional Study | Health Services Research and Managerial Epidemiology | Ethiopia | 2022 | Cross-sectional | Preconception | **Utilization**:   1. Poor knowledge about preconception care 2. Negative attitude towards preconception care |
| 15 | Rakchya Amatya | Factors influencing inequitable access to maternal health services in Morang district, Nepal | Journal of Public Health and Development | Nepal | 2021 | Cross-sectional | Maternal | **Utilization**:   1. Lack of financial accessibility 2. Low autonomy |
| 16 | Banchalem Nega Angore | Determinants of postnatal care utilization in urban community among women in Debre Birhan Town, Northern Shewa, Ethiopia | Journal of Health, Population and Nutrition | Ethiopia | 2018 | Cross-sectional | Postnatal | **Utilization**:   1. Poor knowledge about postnatal   care services   1. Mothers who did not deliver in a health care facility |
| 17 | Judie Arnold | Getting There: Overcoming Barriers to Reproductive and Maternal Health Services Access in Northern Togo—A Qualitative Study | World Medical and Health Policy | Togo | 2016 | Qualitative | Maternal | **Utilization**:   1. Women’s lack of autonomy. 2. Major source of stigmatization for family planning programs. 3. Lack of knowledge regarding contraceptive methods 4. Resultant concerns about potential side effects 5. Distance and the dearth of affordable transportation means   **Provision**:   1. The lack of supplies, 2. Lack of appropriate medicines, 3. Limited space 4. Chastising attitude of health workers |
| 18 | Tsrity Tadese Asresu | Mothers' utilization and associated factors in preconception care in northern Ethiopia: a community based cross sectional study | BMC Pregnancy and Childbirth | Ethiopia | 2019 | Cross sectional | Preconception | **Utilization**:   1. Poor knowledge on preconception care 2. Lack of husband support |
| 19 | Kilian Nasung Atuoye | Maternal health services utilisation among primigravidas in Uganda: what did the MDGs deliver? | Globalization and Health | Uganda | 2020 | Quantitative | Maternal | **Utilization**:   1. Women don’t have decision-making power |
| 20 | Stella Babalola | Determinants of use of maternal health services in Nigeria - Looking beyond individual and household factors | BMC Pregnancy and Childbirth | Nigeria | 2009 | Quantitative | Maternal | **Utilization**:   1. Low community media saturation   **Provision**:   1. Poor ratio of PHC to the population |
| 21 | Stella Babalola | Factors associated with use of maternal health services in Haiti: a multilevel analysis | Rev Panam Salud Publica | Haiti | 2014 | Quantitative | Maternal | **Utilization**:   1. Low community media saturation |
| 22 | Aduragbemi Banke-Thomas | Maternal health services utilisation by Kenyan adolescent mothers: Analysis of the Demographic Health Survey 2014 | Sexual & Reproductive Healthcare | Kenyan | 2017 | Quantitative | Maternal | **Utilization**:   1. Low community media saturation |
| 23 | Roghieh Bayrami | Experiences of women regarding gaps in preconception care services in the Iranian reproductive health care system: A qualitative study | Electronic Physician | Iran | 2016 | Qualitative | Preconception | **Utilization**:   1. Low public awareness of preconception care   **Provision**:   1. Health care providers’ incompetency 2. Lack of integrating preconception care into other health care services 3. Insufficient preconception care package 4. Neglecting adolescent girls in terms of preconception health |
| 24 | Almaz Berhe | Determinants of postnatal care utilization in Tigray, Northern Ethiopia: A community based cross-sectional study | PLOS ONE | Ethiopia | 2019 | Cross-sectional | Postnatal | **Utilization**:   1. Lack of awareness of the services |
| 25 | BhusalCL | Effectiveness and efficiency of Aama Surakshya Karyakram in terms of barriers in accessing maternal health services in Nepal | J Nepal Health Res Counc | Nepal | 2011 | Cross sectional | Maternal | **Utilization**:   1. Lack of transportation facility |
| 26 | Joyce L Browne | Health insurance determines antenatal, delivery and postnatal care utilisation: Evidence from the Ghana Demographic and Health Surveillance data | BMJ Open | Ghana | 2016 | Cross-sectional | Maternal | **Utilization**:  1- Lack of health insurance coverage |
| 27 | Ma. Stephanie Fay S. Cagayan | Barriers to an effective maternal health service delivery network: A qualitative study among health providers in Legazpi City, Albay | Acta Medica Philippina | The Philippines | 2020 | Qualitative | Maternal | **Provision**:   1. Changing political climate. 2. Rapid changes in policies cascaded from the national office. 3. Budget limitations. 4. Lack of and poor implementation of guidelines/protocols 5. Inadequate capacity-building opportunities 6. Poor coordination between facilities 7. Lack of human health resource 8. Poor data management (poor recording and reporting, lack of proper database) 9. Lack of knowledge of role in healthcare teams 10. Unwillingness to learn new skills 11. Competition between public and private healthcare workers |
| 28 | Pooja A. Chauhan | Evaluation of Maternal Health Services Being Provided to the High-risk Mothers of Bhavnagar District, Gujarat | Indian Journal of Community Medicine | India | 2020 | Cross‑sectional | Maternal | **Provision**:   1. 1- Service not offered |
| 29 | Primus Che Chi | A qualitative study exploring the determinants of maternal health service uptake in post-conflict Burundi and Northern Uganda | BMC Pregnancy and Childbirth | Multi-country | 2015 | Qualitative | Maternal | **Utilization**:   1. Lack of women empowerment and support at the household and community   **Provision**:   1. Negative attitude of health providers |
| 30 | Chol Chol | Stakeholders' perspectives on facilitators of and barriers to the utilisation of and access to maternal health services in Eritrea: a qualitative study | BMC Pregnancy and Childbirth | Eritrea | 2018 | Qualitative | Maternal | **Provision**:   1. Lack of ultrasound machines 2. Short clinic opening hours 3. Shortage of healthcare workers 4. Not enough information in the postnatal period 5. Lack of sympathy |
| 31 | Nuzhat Choudhury | Maternal care practices among the ultra-poor households in rural Bangladesh: a qualitative exploratory study | BMC Pregnancy and Childbirth | Bangladesh | 2011 | Qualitative | Maternal | **Utilization**:   1. Hindering cultural beliefs and norms |
| 32 | Rodreck David | Modelling Prenatal Care Pathways at a Central Hospital in Zimbabwe | Health Services Insights | Zimbabwe | 2021 | Quantitative | Prenatal | **Utilization**:   1. Poor family support |
| 33 | Ranjit Kumar Dehury | Health System Competency for Maternal Health Services in Balasore District and Jaleswar Block, Balasore, Odisha, India: An Assessment | Journal of Clinical and Diagnostic Research | India | 2016 | Mixed methods | Maternal | **Utilization**:   1. Poor accessibility through road   Provision:   1. Lack ECG and X-Ray machines for proper diagnostic services. 2. Lack basic diagnostic and ambulance services |
| 34 | Tesfanesh Lemma Demisse | Utilization of preconception care and associated factors among reproductive age group women in Debre Birhan town, North Shewa, Ethiopia. | Reproductive Health | Ethiopia | 2019 | Mixed methods | Preconception | **Utilization**:   1. Poor Knowledge about preconception care   **Provision**:   1. Lack of availability of unit for preconception care |
| 35 | Sulochana Dhakal | Utilisation of postnatal care among rural women in Nepal | BMC Pregnancy and Childbirth | Nepal | 2007 | Cross-sectional | Postnatal | **Utilization**:   1. Lack of awareness among women 2. No antenatal check up 3. Low exposure to mass media 4. Low household autonomy 5. Limited transportation   **Provision**:   1. Lack of trained health workers 2. Lack of health facilities in the village |
| 36 | Farah Diba | Healthcare providers' perception of the referral system in maternal care facilities in Aceh, Indonesia: a cross-sectional study | BMJ Open | Indonesia | 2019 | Cross-sectional | Maternal | **Utilization**:   1. Lack of family consent   **Provision**:   1. Complex administration process |
| 37 | Donmozoun Télesphore Somé | What prevent women for a sustainable use of maternal care in two medical districts of Burkina Faso? A qualitative study | Pan African Medical Journal | Burkina Faso | 2014 | Qualitative | Maternal | **Utilization**:   1. Lack of decision-making power 2. Cultural norms and traditional beliefs 3. Lack of transport means   **Provision**:   1. Bad behavior in maternity services |
| 38 | Kate Doyle | The relationship between inequitable gender norms and provider attitudes and quality of care in maternal health services in Rwanda: a mixed methods study | BMC Pregnancy Childbirth | Rwanda | 2021 | Mixed methods | Maternal | **Provision**:   1. Inequitable gender norms and attitudes of providers |
| 39 | Napoleon N. Ekem | Utilisation of preconception care services and determinants of poor uptake among a cohort of women in Abakaliki Southeast Nigeria | Journal of Obstetrics and Gynaecology | Nigeria | 2018 | Cross-sectional | Preconception | **Utilization**:   1. Low level of awareness |
| 40 | Ghada Wahby Elhady | Postnatal care in rural egypt: Perspectives of women and health care providers | Open Access Macedonian Journal of Medical Sciences | Egypt | 2021 | Cross-sectional | Postnatal | **Utilization**:   1. Perceived incompetence of providers 2. Cultural beliefs 3. Lack of knowledge 4. Dissatisfaction and previous bad experience   **Provision**:   1. Shortage in human resources 2. Lack of logistics 3. Poor Communication of providers 4. Lack of standardized protocol for services |
| 41 | Noureddine Elkhoudri | Postnatal Care: Levels and Determinants in Morocco | Iran J Public Health | Morocco | 2017 | Cross-sectional | Postnatal | **Utilization**:   1. Lack of information   **Provision**:   1. Health professional poor reception |
| 42 | Arone Wondwossen Fantaye | A qualitative study of community elders' perceptions about the underutilization of formal maternal care and maternal death in rural Nigeria | Reproductive Health | Nigeria | 2019 | Qualitative | Maternal | **Utilization**:   1. Lack of community knowledge 2. Transportation barrier 3. Unaffordability   **Provision**:   1. The lack of health professionals 2. Unprofessionalism of health professionals 3. Professionals display patient favoritism 4. Nurses were not following protocol 5. Shortages in medical equipment 6. Shortage in drug supplies. 7. Long wait times in health facilities 8. Provider incompetence 9. Bad Interpersonal relationships between patients and health professionals 10. Absence of a local PHC |
| 43 | Anastasia J. Gage | Effects of the physical accessibility of maternal health services on their use in rural Haiti | Population Studies | Haiti | 2006 | Quantitative | Maternal | **Provision**:   1. Lack of availability of a health center within 5 kilometers |
| 44 | Emebet Gebre | Inequities in maternal health services utilization in Ethiopia 2000-2016: magnitude, trends, and determinants | Reproductive Health | Ethiopia | 2018 | Cross-sectional | maternal | **Utilization**:   1. Fewer accesses to mass media |
| 45 | Genet Gebrehiwot | Postnatal care utilization among urban women in northern Ethiopia: cross-sectional survey | BMC Women's Health | Ethiopia | 2018 | Cross-sectional | Postnatal | **Utilization**:   1. Women who lacked knowledge about the services |
| 46 | Tesfay Gebreslassie Gebrehiwot | Prevalence and associated factors of early postnatal care service use among mothers who had given birth within the last 12 months in Adigrat town, Tigray, northern Ethiopia, 2018 | International Journal of Women’s Health | Ethiopia | 2020 | Cross-sectional | Postnatal | **Utilization**:   1. Low antenatal care visit 2. Awareness on early postnatal care 3. Low prenatal care use |
| 47 | Aklilu Habte | Determinants of practice of preconception care among women of reproductive age group in southern Ethiopia, 2020: content analysis | BMC Reproductive Health | Ethiopia | 2021 | Cross-sectional | Preconception | **Utilization**:   1. Low mother’s knowledge 2. Women’s low autonomy   **Provision**:   1. Lack of availability of service unit |
| 48 | Aklilu Habte | Uptake of complete postnatal care services and its determinants among rural women in Southern Ethiopia: Community-based cross-sectional study based on the current WHO recommendation | PLoS One | Ethiopia | 2021 | Cross-sectional | Postnatal | **Utilization**:   1. Poor knowledge on postnatal care 2. Poor antenatal visits |
| 49 | David R. Hotchkiss | The role of user charges and structural attributes of quality on the use of maternal health services in Morocco | International Journal of Health Planning and Management | Morocco | 2005 | Quantitative | Maternal | **Utilization**:   1. Poor insurance coverage |
| 50 | Xiaohui Hou | The effect of women's decision-making power on maternal health services uptake: evidence from Pakistan | Health Policy and Planning | Pakistan | 2013 | Quantitative | Maternal | **Utilization**:   1. Women’s low decision-making power |
| 51 | Suleman Hadejia Idris | Barriers to utilisation of maternal health services in a semi-urban community in northern Nigeria: The clients' perspective | Nigerian Medical Journal | Nigeria | 2013 | Cross‑sectional | Maternal | **Utilization**:   1. Lack of awareness on existence of postnatal care. 2. Lack of decision-making power by women.   **Provision**:   1. Negative provider attitude 2. Service does not conform with tradition 3. Lack of privacy for staff |
| 52 | Islam MR | Determinants of antenatal and postnatal care visits among Indigenous people in Bangladesh: a study of the Mru community | Rural Remote Health | Bangladesh | 2011 | Cross-sectional | Maternal | **Utilization**:   1. Using traditional practices 2. Transportation problems 3. Low exposure to mass media |
| 53 | C. Kambala | Barriers to maternal health service use in Chikhwawa, southern Malawi | Malawi Medical Journal | Malawi | 2011 | Qualitative | Maternal | **Utilization**:   1. Lack of decision-making power 2. Using traditional services   **Provision**:   1. Rude health staff 2. Lack of mid-wives 3. Lack of or insufficient items 4. Long stay in the hospital |
| 54 | Sumit Kane | Too afraid to go: fears of dignity violations as reasons for non-use of maternal health services in South Sudan | Reproductive Health | South Sudan | 2018 | Qualitative | Maternal | **Utilization**:   1. Fear of experiencing dignity violations 2. Fear of being embarrassed 3. Fear of being ill-treated 4. Insecurity related fear |
| 55 | Andargachew Kassa | Level of Healthcare Providers' Preconception Care (PCC) Practice and Factors Associated with Non-Implementation of PCC in Hawassa, Ethiopia | Ethiop J Health Sci | Ethiopia | 2019 | Cross-sectional | Preconception | **Provision**:   1. Providers’ Poor preconception care Knowledge |
| 56 | Aschenaki Z. Kea | Exploring barriers to the use of formal maternal health services and priority areas for action in Sidama zone, southern Ethiopia | BMC Pregnancy and Childbirth | Ethiopia | 2018 | Qualitative | Maternal | **Utilization**:   1. Lack of Knowledge 2. Bad previous experience 3. Traditional beliefs 4. Lack of decision-making power 5. Non familiarity with setting 6. Perception of lack of privacy 7. Poor transportation   **Provision**:   1. Shortage of equipment 2. Lack of proper referral 3. Bad behavior of provider 4. Lack of knowledge 5. Lack of skill |
| 57 | Raz Mohammad Khan Khankhell | Factors influencing utilization of postnatal care visits in Afghanistan | Nagoya Journal of Medical Science | Afghanistan | 2020 | Cross-sectional | Postnatal | **Utilization**:   1. Low antenatal care 2. Low exposure to public media 3. Woman have not role in decision making |
| 58 | Stephen M. Kibusi | Health insurance is important in improving maternal health service utilization in Tanzania - Analysis of the 2011/2012 Tanzania HIV/AIDS and malaria indicator survey | BMC Health Services Research | Tanzania | 2018 | Quantitative | Maternal | **Utilization**:   1. Low coverage of health insurance |
| 59 | Eunsoo Timothy Kim | Availability of health facilities and utilization of maternal and newborn postnatal care in rural Malawi | BMC Pregnancy and Childbirth | Malawi | 2019 | Quantitative | Postnatal | **Utilization**:   1. Great distance from health facilities |
| 60 | Rosemary King | Utilisation of maternal health services in Ethiopia: a key informant research project | Development in Practice | Ethiopia | 2016 | Qualitative | Perinatal | **Utilization**:   1. Lack of available transport 2. Lack of awareness about health services 3. Lack of awareness of need 4. Cultural preference 5. Need for husband’s permission   **Provision**:   1. Lack of service 2. Limited opening hours 3. Poor staff training 4. Lack of staff 5. Limited resources 6. Staffs' bad attitudes and interpersonal skills |
| 61 | Sue King | Perceptions and use of maternal health services by women in rural coastal Madang Province | Papua and New Guinea medical journal | Papua New Guinea | 2013 | Cross-sectional | Maternal | **Provision**:   1. Bad attitude of providers 2. Lack of services |
| 62 | Eveline T. Konje | Late initiation and low utilization of postnatal care services among women in the rural setting in Northwest Tanzania: a community-based study using a mixed method approach | BMC Health Services Research | Tanzania | 2021 | Mixed methods | Postnatal | **Utilization**:   1. Limited knowledge on the postnatal care services 2. Not being scheduled for postnatal care by health providers   **Provision**:   1. Overwhelming workload 2. Shortages of supplies |
| 63 | Lily Kumbani | Why some women fail to give birth at health facilities: a qualitative study of women's perceptions of perinatal care from rural Southern Malawi | Reproductive Health | Malawi | 2013 | Qualitative | Perinatal | **Utilization**:   1. Low access   **Provision**:   1. Long waiting time 2. Health workers were rude 3. Low confidentiality |
| 64 | Miteku Andualem Limenih | Postnatal Care Service Utilization and Associated Factors among Women Who Gave Birth in the Last 12 Months prior to the Study in Debre Markos Town, Northwestern Ethiopia: A Community-Based Cross-Sectional Study | International Journal of Reproductive Medicine | Ethiopia | 2016 | Cross-Sectional | Postnatal | **Utilization**:   1. Low awareness about maternal complication |
| 65 | Kennedy Machira | Rural-urban differences in the use of postnatal care services in Malawi | J Egypt Public Health Assoc | Malawi | 2017 | Cross-Sectional | Postnatal | **Utilization**:   1. Low media exposure 2. Poor transportation   **Provision**:   1. Non availability of health services |
| 66 | Eric M. Mafuta | Factors influencing the capacity of women to voice their concerns about maternal health services in the Muanda and Bolenge Health Zones, Democratic Republic of the Congo: a multi-method study | BMC Health Services Research | Congo | 2018 | Quantitative | Maternal | **Utilization:**   1. Women were unaware of their entitlements and rights |
| 67 | Marshall Makate | Prenatal care utilization in Zimbabwe: Examining the role of community-level factors | Journal of Epidemiology and Global Health | Zimbabwe | 2017 | Cross-sectional | Prenatal | **Utilization:**   1. Low usage of mass media 2. Low insurance coverage |
| 68 | Fredrick Manang | The impact of access to health facilities on maternal care use, travel patterns, and health status: Evidence from longitudinal data from Uganda | Economic Development and Cultural Change | Uganda | 2020 | Quantitative | Maternal | **Provision:**   1. Lack of availability of facilities |
| 69 | Markos Manote | Determinants of postnatal care non-utilization among women in Demba Gofa rural district, southern Ethiopia: a community-based unmatched case-control study | BMC Pregnancy and Childbirth | Ethiopia | 2020 | Case-control | Postnatal | **Utilization:**   1. Not knowing the availability of PNC services 2. Antenatal care non-attendance 3. Unable to make an independent decision |
| 70 | Adine Marquis | Use of and barriers to maternal health services in southeast Chad: results of a population-based survey 2019 | BMJ Open | Chad | 2021 | Cross-sectional | Maternal | **Utilization:**   1. Low use of antenatal care 2. Poor transportation 3. Cultural beliefs 4. Lack of family support |
| 71 | Masaki Matsumura | Women's status, household structure and the utilization of maternal health services in Nepal | Asia-Pacific Population Journal | Nepal | 2001 | Quantitative | Maternal | **Utilization:**   1. Low decision-making power |
| 72 | Sadatoshi Matsuoka | Perceived barriers to utilization of maternal health services in rural Cambodia | Health Policy | Cambodia | 2010 | Qualitative | Maternal | **Utilization:**   1. Beliefs about the quality of health services 2. Limited knowledge or misinformation about health professionals and services 3. The perceived limited midwifery skills 4. Experiential judgment of the elderly of the community 5. Traditional beliefs and practices 6. Lack of confidence in providers   **Provision:**   1. Lack of availability of services 2. Lack of ward space for resting after delivery 3. Impolite behavior of health professionals 4. Absence of health staff |
| 73 | Vernon Mochache | Religious, socio-cultural norms and gender stereotypes influence uptake and utilization of maternal health services among the Digo community in Kwale, Kenya: a qualitative study | Reproductive Health | Kenya | 2020 | Qualitative | Maternal | **Utilization:**   1. Dominance of cultural beliefs 2. Dominance of traditional health workers 3. Lack of decision-making power |
| 74 | Mohamed-Ahmed R | Antenatal care in Sudan: A qualitative study into accessibility and quality of maternal health services in Khartoum | International Journal of Childbirth | Sudan | 2018 | Qualitative | Maternal | **Utilization:**   1. Provider’s perceived lack of empathy 2. Lack of health advice 3. Dissatisfied with the quality of care |
| 75 | Aye Sandar Mon | Utilization of full postnatal care services among rural Myanmar women and its determinants: a cross-sectional study | F1000Research | Myanmar | 2018 | Cross-sectional | Postnatal | **Utilization:**   1. Low awareness of postnatal danger signs 2. Misconceptions on postnatal practice |
| 76 | Rose NM Mpembeni | Use pattern of maternal health services and determinants of skilled care during delivery in Southern Tanzania: implications for achievement of MDG-5 targets | BMC Pregnancy and Childbirth | Tanzania | 2007 | Cross-sectional | Maternal | **Utilization:**   1. Low knowledge of pregnancy risk factors   **Provision:**   1. Lack of availability of services |
| 77 | Mwifadhi Mrisho | The use of antenatal and postnatal care: perspectives and experiences of women and health care providers in rural southern Tanzania | BMC Pregnancy and Childbirth | Tanzania | 2009 | Qualitative | Perinatal | **Utilization:**   1. Fear of caesarean 2. Shyness or embarrassment   **Provision:**   1. Lack of availability of services |
| 78 | Ngatho S. Mugo | Barriers Faced by the Health Workers to Deliver Maternal Care Services and Their Perceptions of the Factors Preventing Their Clients from Receiving the Services: A Qualitative Study in South Sudan | Maternal and Child Health Journal | Sudan | 2018 | Qualitative | Maternal | **Utilization**:   1. Poor transportation 2. Lack of awareness of benefit of services 3. Lack of family support 4. Traditional belief   **Provision**:   1. Lack of facility 2. Long waiting time 3. Poor quality of services 4. Lack of incentive among providers 5. Low level of skill 6. Lack of enough staff 7. Insufficient training 8. Lack of managerial coordination 9. Lack of drug and medical supply 10. Lack of equipment 11. Limited spaces 12. Poor infrastructures |
| 79 | THT Nguyen | Health Workers' Perspectives on Infrastructure to Support Maternal Health Services in Rural Areas of Vietnam | Asia Pacific Journal of Health Management | Vietnam | 2017 | Qualitative | Maternal | **Provision:**   1. Small Size of facilities 2. Lack of a permanent building |
| 80 | Chinomnso C Nnebue | Adequacy of resources for provision of maternal health services at the primary health care level in Nnewi, Nigeria | Nigerian Medical Journal | Nigeria | 2014 | Cross-sectional | Maternal | **Provision:**   1. Insufficient equipment 2. Insufficient drugs 3. Insufficient staff |
| 81 | Chinomnso C. Nnebue | Availability and Continuity of Care for Maternal Health Services in the Primary Health Centres in Nnewi, Nigeria (January - March 2010) | International Journal of Preventive Medicine | Nigeria | 2016 | Cross‑sectional | Maternal | **Provision:**   1. Lack of drugs 2. Lack of equipment 3. Lack of skilled personnel 4. Lack of a standardized protocol for referring clients |
| 82 | Lorretta Favour C. Ntoimo | Why rural women do not use primary health centres for pregnancy care: Evidence from a qualitative study in Nigeria | BMC Pregnancy and Childbirth | Nigeria | 2019 | Qualitative | Prenatal | **Utilization:**   1. Poor transportation 2. Informal payments 3. Lack of family support 4. Misinterpretation of signs of pregnancy complications   **Provision:**   1. Lack of availability of services 2. Facility not always open 3. Low Provider competence 4. Unfriendly attitude of providers 5. Inadequate providers 6. Long waiting time 7. Inappropriate referral 8. Inadequate supply of drugs 9. Bad environment of facilities |
| 83 | Dewi Nuryana | Maternal health services utilization and its contributing factors among adolescent mothers | International Journal of Public Health Science | Indonesia | 2022 | Quantitative | Maternal | **Utilization:**   1. Low mother’s autonomy 2. Limited knowledge of danger sign 3. Low coverage of health insurance |
| 84 | Angelo S. Nyamtema | Dar es Salaam perinatal care study: needs assessment for quality of care | East African Journal of Public Health | Tanzania | 2008 | Cross-sectional | Perinatal | **Provision:**   1. Inadequate infrastructure 2. Inadequate equipment and supplies |
| 85 | Titilayo Dorothy Odetola | Assessment of Perinatal Care Satisfaction Amongst Mothers Attending Postnatal Care in Ibadan, Nigeria | Annals of Global Health | Nigeria | 2018 | Cross-sectional | Perinatal | **Provision:**   1. Dirty hospital environment 2. Inadequate infrastructures 3. Long waiting time 4. Inadequate staffing 5. Poor staff attitude 6. Poor staff behavior |
| 86 | Oludoyinmola O. Ojifinni | Exploring the perception of and attitude towards preconception care service provision and utilisation in a south western nigerian community – a qualitative study | Etude de la Population Africaine | Nigeria | 2021 | Qualitative | Preconception | **Utilization:**   1. Cultural belief 2. Lack of awareness of service benefits 3. Preference for traditional medicine |
| 87 | J. K. Okemo | Determinants of preconception care among pregnant women in an urban and a rural health facility in Kenya: a qualitative study | BMC Pregnancy and Childbirth | Kenya | 2021 | Qualitative | Preconception | **Utilization:**   1. Lack of awareness about services 2. Personal attitude toward care 3. Previous bad interactions with health professionals 4. Low exposure to social media   **Provision:**   1. Lack of availability of services |
| 88 | Friday Okonofua | Predictors of women's utilization of primary health care for skilled pregnancy care in rural Nigeria | BMC Pregnancy and Childbirth | Nigeria | 2018 | Cross-sectional | Prenatal | **Utilization**:   1. Low autonomy 2. Poor transportation 3. Preference of traditional medicine 4. Cultural belief   **Provision**:   1. Lack of availability of services 2. Poor quality of PHC service delivery 3. Staff shortage |
| 89 | Peter Oyovwe | Exploring health care professionals' and women's perspectives on the barriers to maternal health services: a qualitative study in Eku Town of Delta State, Nigeria | AIMS Public Health | Nigeria | 2021 | Qualitative | Maternal | **Utilization**:   1. Cultural belief 2. Low awareness   **Provision**:   1. Negative attitude of providers |
| 90 | Parisa Patel | The perceptions, health-seeking behaviours and access of Scheduled Caste women to maternal health services in Bihar, India | Reproductive Health Matters | India | 2018 | Qualitative | Maternal | **Utilization:**   1. Poor insurance coverage 2. Poor knowledge on services 3. Cultural belief   **Provision:**   1. Shortage of healthcare staff 2. Lack of availability of services |
| 91 | Sapnaben Bhavin Patel | A mixed method study on utilization of maternal health services and barriers among women of reproductive age in Gujarat state-pilot study | Indian Journal of Public Health Research and Development | India | 2018 | Mixed methods | Maternal | **Utilization**:   1. Cultural and traditional belief   **Provision**:   1. Shortage of staff 2. Bad clinic environment 3. Long waiting time 4. Limited hour access to services |
| 92 | Karen Odberg Pettersson | Mozambican midwives' views on barriers to quality perinatal care | Health Care for Women International | Mozambic | 2006 | Qualitative | Perinatal | **Provision**:   1. Inadequate staff 2. Inadequate equipment 3. Inadequate space 4. Poor referral system 5. Lack of competency 6. Non-appliance of Best Caring Practices |
| 93 | Mercy Pindani | Health Surveillance Assistants' Practices of Postnatal Care in Lilongwe District, Malawi | Africa Journal of Nursing and Midwifery | Malawi | 2020 | Mixed methods | Postnatal | **Provision:**   1. lack of knowledge 2. lack of skills |
| 94 | Ari Probandari | Barriers to utilization of postnatal care at village level in Klaten district, central Java Province, Indonesia | BMC Health Services Research | Indonesia | 2017 | Qualitative | Postnatal | **Utilization**:   1. Lack of Knowledge on danger signs 2. Lack of health knowledge 3. Cultural belief 4. Language barrier 5. Self-treatment and traditional care 6. Lack of decision power   **Provision**:   1. Shortage of staff 2. Low quality services |
| 95 | Anissa Rizkianti | Women's decision-making autonomy in the household and the use of maternal health services: An Indonesian case study | Midwifery | Indonesia | 2020 | Quantitative | Maternal | **Utilization**:   1. Low decision-making autonomy |
| 96 | Emma Sacks | Postnatal Care Experiences and Barriers to Care Utilization for Home- and Facility-Delivered Newborns in Uganda and Zambia | Matern Child Health J | Multi national | 2017 | Qualitative | Postnatal | **Utilization**:   1. Lack of Importance 2. Fear of Mistreatment by Clinic Staff 3. Negative Postnatal Care Experiences |
| 97 | Mariyam Sarfraz | Challenges in delivery of skilled maternal care - experiences of community midwives in Pakistan | BMC Pregnancy and Childbirth | Pakistan | 2104 | Qualitative | Maternal | **Provision**:   1. Inadequately trained staff 2. Lack of sufficient resources 3. Lack of integration in district health system |
| 98 | Nirmal Shahzaib | Factors affecting Maternal-care during labour at maternity centres of Karachi, Pakistan: Exploratory study | Journal of the Pakistan Medical Association | Pakistan | 2021 | Qualitative | Maternal | **Provision**:   1. Unhygienic environment 2. Lack of basic equipment and supplies 3. Lack of medicine 4. Unprofessional attitude of staff 5. Poor physical infrastructure 6. Shortage of staff 7. Lack of guidelines 8. Poor planning 9. Low new staff knowledge |
| 99 | Dipa Sharma Gautam | No time, no money, no luck: Barriers to prenatal care among dalit women in rural Nepal | Health Care for Women International | Nepal | 2019 | Qualitative | Prenatal | **Utilization**:   1. Cultural belief 2. Low decision-making power |
| 100 | Abhishek Singh | A cross-sectional study to assess the utilization pattern of maternal health services and associated factors in aspirational district of Haryana, India | Journal of Family Medicine and Primary Care | India | 2021 | Cross-sectional | Maternal | **Utilization:**   1. Low decision‑making capacity |
| 101 | Nighat Sultana | Low utilization of postnatal care: searching the window of opportunity to save mothers and newborns lives in Islamabad capital territory, Pakistan | BMC Research Note | Pakistan | 2015 | Cross-sectional | Postnatal | **Utilization**:   1. Lack of awareness about benefit of services 2. Lack of decision‑making power 3. Poor transportation 4. Lack of knowledge about available services   **Provision**:   1. Lack of availability of services |
| 102 | Fikirte Tesfahun | Knowledge, perception and utilization of postnatal care of mothers in Gondar Zuria District, Ethiopia: a cross-sectional study | Matern Child Health J | Ethiopia | 2014 | Cross-Sectional | Postnatal | **Utilization**:   1. Poor antenatal care visit 2. Low decision-making authority   **Provision**:   1. Lack of service |
| 103 | Gezahegn Tesfaye | Delaying factors for maternal health service utilization in eastern Ethiopia: A qualitative exploratory study | Women and Birth | Ethiopia | 2020 | Qualitative | Maternal | **Utilization**:   1. Poor knowledge about the important of service 2. Cultural restrictions 3. Poor transportation 4. Negative attitudes towards male midwives 5. Acceptance of traditional birth attendants 6. Low health knowledge   **Provision**:   1. Poor quality of care |
| 104 | Firanbon Teshome | Why do women not prepare for pregnancy? Exploring women's and health care providers' views on barriers to uptake of preconception care in Mana District, Southwest Ethiopia: a qualitative study | BMC Pregnancy and Childbirth | Ethiopia | 2020 | Qualitative | Preconception | **Utilization**:   1. Lack of knowledge about care 2. Traditional belief 3. Low decision-making power 4. Low media information   **Provision**:   1. Lack of availability of services |
| 105 | Christiana R Titaley | Why don't some women attend antenatal and postnatal care services? a qualitative study of community members' perspectives in Garut, Sukabumi and Ciamis districts of West Java Province, Indonesia | BMC Pregnancy and Childbirth | Indonesia | 2010 | Qualitative | Perinatal | **Utilization**:   1. Lack of community awareness about the importance of these services 2. Preference for traditional services   **Provision**:   1. Limited availability of health services |
| 106 | Habtamu Tolera | Risk factors for women's non-utilization of decentralized primary health care facilities for postnatal care in rural western Ethiopia | Therapeutic Advances in Reproductive Health | Ethiopia | 2020 | Quantitative | Postnatal | **Utilization**:   1. Not receiving antenatal care 2. No knowledge of at least one postnatal complication 3. No knowledge about the availability of services |
| 107 | Berhan Tsegaye | Prevalence and Factors Associated with Immediate Postnatal Care Utilization in Ethiopia: Analysis of Ethiopian Demographic Health Survey 2016 | International Journal of Women’s Health | Ethiopia | 2021 | Quantitative | Postnatal | **Utilization:**   1. Knowledge about pregnancy complication 2. Low use of media |
| 108 | Berhan Tsegaye | Predictors of skilled maternal health services utilizations: A case of rural women in Ethiopia | PLoS One | Ethiopia | 2021 | Cross-sectional | Maternal | **Utilization:**   1. Low antenatal care 2. Low information about postnatal care service utilization 3. Low women autonomy |
| 109 | Ogochukwu Udenigwe | Perspectives of policymakers and health providers on barriers and facilitators to skilled pregnancy care: findings from a qualitative study in rural Nigeria | BMC Pregnancy and Childbirth | Nigeria | 2021 | Qualitative | Prenatal | **Utilization**:   1. Lack of decision-making power 2. Cultural belief 3. Poor knowledge on benefits of services 4. Language barrier 5. Lack of social support 6. Previous negative experience with skilled healthcare   **Provision**:   1. Lack of availability of services 2. Shortage of skilled health personnel 3. Poor infrastructures 4. Shortage of medical equipment |
| 110 | Neha Upadhyai | Utilization of postnatal care services and factors affecting it among women of urban slums in Dehradun, Uttarakhand | Indian Journal of Community Health | India | 2019 | Cross-sectional | Postnatal | **Utilization**:   1. Low perception of health problem 2. Low antenatal care |
| 111 | Meron Admasu Wegene | Utilization of preconception care and associated factors in Hosanna Town, Southern Ethiopia | PLoS One | Ethiopia | 2022 | Cross-sectional | Preconception | **Utilization**:   1. Poor knowledge on preconception 2. Not receiving counseling on preconception care previously |
| 112 | Berhanu Teshome Woldeamanuel | Trends, regional inequalities and determinants in the utilization of prenatal care and skilled birth attendant in Ethiopia: A multilevel analysis | Clinical Epidemiology and Global Health | Ethiopia | 2021 | Quantitative | Prenatal | **Utilization**:   1. Low decision-making power 2. Low insurance coverage   **Provision**:   1. Lack of availability of services |
| 113 | Vida Nyagre Yakong | Women's experiences of seeking reproductive health care in rural Ghana: Challenges for maternal health service utilization | Journal of Advanced Nursing | Ghana | 2010 | Qualitative | Maternal | **Provision**:   1. Bad providers behavior 2. Lack of patient education by staff 3. Limited care choices 4. Limited space |
| 114 | Sanni Yaya | Gender inequity as a barrier to women's access to skilled pregnancy care in rural Nigeria: A qualitative study | International Health | Nigeria | 2019 | Qualitative | Prenatal | **Utilization**:   1. Low decision-making power |
| 115 | Gloria-Sheila A. Yiran | Accessibility and Utilisation of Maternal Health Services by Migrant Female Head Porters in Accra | Journal of International Migration and Integration | Ghana | 2015 | Quantitative | Maternal | **Utilization**:   1. Preference for traditional medicine   **Provision**:   1. Long waiting time |
| 116 | Shambel Yoseph | Prevalence of Early Postnatal-Care Service Utilization and Its Associated Factors among Mothers in Hawassa Zuria District, Sidama Regional State, Ethiopia: A Cross-Sectional Study | Obstetrics and Gynecology International | Ethiopia | 2021 | Cross-Sectional | Postnatal | **Utilization:**   1. Low information about obstetric danger signs 2. Having negative attitude on use postnatal services |
| 117 | Liknaw Bewket Zeleke | Postnatal care service utilization and its determinants in East Gojjam Zone, Northwest Ethiopia: A mixed-method study | PLoS One | Ethiopia | 2021 | Mixed-methods | Postnatal | **Utilization**:   1. Not aware of the services 2. Lack of family support 3. Traditional beliefs 4. Transportation problems   **Provision**:   1. Long waiting time 2. Lack of availability of services |
