# Supplementary material for: Key barriers to the provision and utilization of maternal health services in low-and lower-middle-income countries; a scoping review
Source: BMC Womens Health. 2024 Jun 5;24:325. doi: 10.1186/s12905-024-03177-x (PMC11151574; doi:10.1186/s12905-024-03177-x)
Supplement: Supplementary file 2 — Supplementary Material 2. [file 12905_2024_3177_MOESM2_ESM.docx]

**Challenges in the provision of maternal health Services**

**Thematic Analysis (Initial codes & categories)**

| **No** | **Categories** | **Initial Codes (Article number in data charting form)** |
| --- | --- | --- |
| 1 | Shortage of equipment | Lack of medical equipment (7)  The lack of supplies (17)  Lack of ultrasound machines (30)  Lack of logistics (40)  Shortages in medical equipment (42)  Lack of or insufficient items (53)  Shortage of equipment (56)  Shortages of supplies (62)  Lack of equipment (78)  Insufficient equipment (80)  Lack of equipment (81)  Inadequate equipment and supplies (84)  Inadequate equipment (92)  Lack of basic equipment and supplies (98)  Shortage of medical equipment (109) |
| 2 | Shortage of medicine | Lack of availability of drugs (7)  Lack of appropriate medicines (17)  Shortage in drug supplies (42)  Lack of drug (78)  Insufficient drugs (80)  Lack of drugs (81)  Inadequate supply of drugs (82)  Lack of medicine (98) |
| 3 | Limited hours of service | Centers were always locked, especially at night (7)  Short clinic opening hours (30)  Limited opening hours (60)  Absence of health staff (72)  Facility not always open (82)  Limited hour access to services (91) |
| 4 | Limitations of service delivery space | Limited space (17)  Lack of privacy for staff (51)  Lack of ward space for resting (72)  Limited spaces (78)  Small Size of facilities (79)  Inadequate space (92)  Limited space (113) |
| 5 | Negative attitude of the health workforce | Chastising attitude of health workers (17)  Negative attitude of health providers (29)  Lack of sympathy (30)  Bad behavior in maternity services (37)  Inequitable gender norms and attitudes of providers (38)  Poor Communication of providers (40)  Health professional poor reception (41)  Professionals display patient favoritism (42)  Bad Interpersonal relationships between patients and health professionals (42)  Unprofessionalism of health professionals (42)  Negative provider attitude (51)  Rude health staff (53)  Bad behavior of provider (56)  Staffs' bad attitudes and interpersonal skills (60)  Bad attitude of providers (61)  Health workers were rude (63)  Low confidentiality (63)  Impolite behavior of health professionals (72)  Unfriendly attitude of providers (82)  Poor staff attitude (85)  Poor staff behavior (85)  Negative attitude of providers (89)  Unprofessional attitude of staff (98)  Bad providers behavior (113) |
| 6 | Incompetence of health professionals | Health care providers’ incompetency (23)  Inadequate capacity-building opportunities (27)  Unwillingness to learn new skills (27)  Provider incompetence (42)  Lack of skill (56)  Low level of skill (78)  Low Provider competence (82)  Lack of competency (92)  Lack of skills (92) |
| 7 | Lack of integrity of the service delivery system | Lack of integrating preconception care into other health care services (23)  Lack of proper referral (56)  Lack of a standardized protocol for referring clients (81)  Inappropriate referral (82)  Poor referral system (92)  Lack of integration in district health system (97) |
| 8 | Insufficient infrastructure | Poor infrastructures (78)  Inadequate infrastructure (84)  Inadequate infrastructures (85)  Poor physical infrastructure (98)  Poor infrastructures (109) |
| 9 | Limitation of resources | Budget limitations (27)  Limited resources (60)  Lack of sufficient resources (97) |
| 10 | Shortage of physical capital | Lack of a permanent building (79) |
| 11 | Weakness of managerial processes | Poor coordination between facilities (27)  Complex administration process (36)  Lack of managerial coordination (78)  Poor planning (98) |
| 12 | Shortage of human resources | Lack of human health resource (27)  Shortage of healthcare workers (30)  Lack of trained health workers (35)  Shortage in human resources (40)  The lack of health professionals (42)  Lack of mid-wives (53)  Lack of staff (60)  Overwhelming workload (62)  Lack of enough staff (78)  Insufficient staff (80)  Lack of skilled personnel (81)  Inadequate providers (82)  Inadequate staffing (85)  Staff shortage (88)  Shortage of healthcare staff (90)  Shortage of staff (91)  Inadequate staff (92)  Shortage of staff (95)  Shortage of staff (98)  Shortage of skilled health personnel (109) |
| 13 | Lack of availability of services | Insufficient preconception care package (23)  Service not offered (28)  Lack of availability of unit for preconception care (34)  Lack of health facilities in the village (35)  Absence of a local PHC (42)  Lack of availability of a health center within 5 kilometers (43)  Lack of availability of service unit (47)  Lack of service (60)  Lack of services (61)  Non availability of health services (65)  Lack of availability of facilities (68)  Lack of availability of services (72)  Lack of availability of services (76)  Lack of availability of services (77)  Lack of facility (78)  Lack of availability of services (82)  Lack of availability of services (87)  Lack of availability of services (88)  Lack of availability of services (90)  Lack of availability of services (101)  Lack of service (102)  Lack of availability of services (104)  Limited availability of health services (105)  Lack of availability of services (109)  Lack of availability of services (112)  Limited care choices (113)  Lack of availability of services (117) |
| 14 | Inappropriate service delivery environment | Non-clean environment (11)  Bad environment of facilities (82)  Dirty hospital environment (85)  Bad clinic environment (91)  Unhygienic environment (98) |
| 15 | Lack of appropriate guidelines | Neglecting adolescent girls in terms of preconception health (23)  Lack of standardized protocol for services (40)  Nurses were not following protocol (42)  Non-appliance of Best Caring Practices (92)  Lack of guidelines (98) |
| 16 | Long waiting list | Long wait times in health facilities (42)  Long stay in the hospital (53)  Long waiting time (63)  Long waiting time (78)  Long waiting time (82)  Long waiting time (85)  Long waiting time (91)  Long waiting time (115)  Long waiting time (117) |
| 17 | Inadequate knowledge of service providers | Lack of knowledge of role in healthcare teams (27)  Not enough information in the postnatal period (30)  Providers’ Poor preconception care Knowledge (55)  Lack of knowledge (56)  Poor staff training (60)  Insufficient training (78)  Lack of knowledge (92)  Inadequately trained staff (97)  Low new staff knowledge (98) |
| 18 | Poor quality of service | Service does not conform with tradition (51)  Poor quality of services (78)  Poor quality of PHC service delivery (88)  Low quality services (95)  Poor quality of care (103)  Lack of patient education by staff (113) |
| 19 | Insufficient motivation of service providers | Competition between public and private healthcare workers (27)  Lack of incentive among providers (78) |
| 20 | Poor management of the information system | Poor data management (27) |
| 21 | Political restrictions | Changing political climate (27)  Rapid changes in policies cascaded from the national office (27) |

**Challenges in the provision of maternal health Services**

**Thematic Analysis (Categories, sub-themes & themes)**

| **Themes** | **Sub-themes** | **No** | **Categories** |
| --- | --- | --- | --- |
| Resource, equipment, and capital constraints | Shortage of medical equipment and supplies | 1 | Shortage of equipment |
|  |  | 2 | Shortage of medicine |
|  | Restrictions on the physical space of the services | 4 | Limitations of service delivery space |
|  |  | 8 | Insufficient infrastructure |
|  |  | 14 | Inappropriate service delivery environment |
|  | Limitation of resources | 9 | Limitation of resources |
|  |  | 10 | Shortage of physical capital |
| Human resource barriers | Shortage of health workforce | 3 | Limited hours of service |
|  |  | 12 | Shortage of human resources |
|  | Weakness of scientific and  practical capabilities of health workforce | 5 | Negative attitude of the health workforce |
|  |  | 6 | Incompetence of health professionals |
|  |  | 17 | Inadequate knowledge of service providers |
|  |  | 19 | Insufficient motivation of service providers |
| process defects in the provision of services | Challenges in providing standards-compliant services | 15 | Lack of appropriate guidelines |
|  |  | 18 | Poor quality of service |
|  | Defects in the service management system | 7 | Lack of integrity of the service delivery system |
|  |  | 11 | Weakness of managerial processes |
|  |  | 20 | Poor management of the information system |
|  |  | 21 | Political restrictions |
|  | Weakness in providing adequate essential services | 13 | Lack of availability of services |
|  |  | 16 | Long waiting list |

**Challenges in the utilization of Maternal Health Services**

**Thematic Analysis (Initial codes & categories)**

| **No** | **Categories** | **Initial Codes (Article’s number in data charting form)** |
| --- | --- | --- |
| 1 | Lack of awareness of risk factors and danger signs | Didn’t aware of at least one postpartum danger signs (1)  Low awareness about problems of postnatal period (3)  Poor knowledge of delivery-related complications (6)  Having knowledge about pregnancy complications (13)  LOW awareness about maternal complication (64)  Low awareness of postnatal danger signs (75)  Low knowledge of pregnancy risk factors (76(  Misinterpretation of signs of pregnancy complications (82)  Limited knowledge of danger signs (83)  Lack of Knowledge on danger signs (94)  No knowledge of at least one postnatal complication (106)  Knowledge about pregnancy complication (107)  Low information about obstetric danger signs (116) |
| 2 | Lack of awareness of available maternal health services | Not aware where to go for pregnancy complications (12)  Poor knowledge about preconception care (14)  Poor knowledge about postnatal care services (16)  Lack of knowledge regarding contraceptive methods (17)  Poor knowledge on preconception care (18)  Low public awareness of PCC (23)  Lack of awareness of the services (24)  Poor Knowledge about preconception care (34)  Lack of awareness among women (35)  Low level of awareness (39)  Lack of knowledge (40)  Lack of information (41)  Lack of community knowledge (42)  Women who lacked knowledge about the services (45)  Awareness on early postnatal care (46)  Low mother’s knowledge (47)  Poor knowledge on postnatal care (48)  Lack of awareness on existence of postnatal care (51)  Lack of Knowledge (56)  Non familiarity with setting (56)  Lack of awareness about health services (60)  Limited knowledge on the postnatal care services (62)  Not knowing the availability of PNC services (69)  Limited knowledge or misinformation about health services (72)  Lack of health advice (74)  Misconceptions on postnatal practice (75)  Lack of awareness of benefit of services (78)  Lack of awareness of service benefits (86)  Lack of awareness about services (87)  Low awareness (89)  Poor knowledge on services (90)  Lack of health knowledge (94)  Lack of awareness about benefit of services (101)  Lack of knowledge about available services (101)  Low health knowledge (103)  Poor knowledge about the important of service (103)  Lack of knowledge about care (104)  Lack of community awareness about the importance of these services (105)  No knowledge about the availability of services (106)  Low information about postnatal care service utilization (108)  Poor knowledge on benefits of services (109)  Low perception of health problem (110)  Poor knowledge on preconception (111)  Not aware of the services (117) |
| 3 | Failure to receive special care and information in previous stages of care | Not used antenatal care (2)  Low Rate of antenatal care follow-up (3)  Delayed antenatal care (8)  Mothers who did not deliver in a health care facility (16)  No antenatal checkup (35)  Low antenatal care visits (46)  Low postnatal care use (46)  Poor antenatal visits (48)  Low antenatal care (57)  Not being scheduled for postnatal care by health providers (62)  Antenatal care non-attendance (69)  Low use of antenatal care (70)  Poor antenatal care visits (102)  Not receiving antenatal care (106)  Low antenatal care (108)  Low antenatal care (110)  Not receiving counseling on preconception care previously (111) |
| 4 | Poor insurance coverage | Lack of health insurance coverage (4)  Lack of health insurance coverage (26)  Poor insurance coverage (49)  Low coverage of health insurance (58)  Low insurance coverage (67)  Low coverage of health insurance (83)  Poor insurance coverage (90)  Low insurance coverage (112) |
| 5 | limited autonomy and low decision-making power | Low level of autonomy (5)  Women’s lack of opportunity to make decision (9)  Woman's freedom of movement (13)  Low autonomy (15)  Women’s lack of autonomy (17)  Women don’t have decision-making power (19)  Low household autonomy (35)  Lack of decision-making power (37)  Women’s low autonomy (47)  Women’s low decision-making power (50)  Lack of decision-making power by women (51)  Lack of decision-making power (53)  Lack of decision-making power (56)  Woman have not role in decision making (57)  Women were unaware of their entitlements and rights (66)  Unable to make an independent decision (69)  Low decision-making power (71)  Lack of decision-making power (73)  Low mother’s autonomy (83)  Low autonomy (88)  Lack of decision power (94)  Low decision-making autonomy (95)  Low decision-making power (99)  Low decision‑making capacity (100)  Lack of decision‑making power (101)  Low decision-making authority (102)  Low decision-making power (104)  Low women autonomy (108)  Lack of decision-making power (109)  Low decision-making power (112)  Low decision-making power (114) |
| 6 | Dependence on spouse in decision-making | Women were submissive to their husbands (7)  No permission to go to hospital without a guardian (9)  Need for husband’s permission (60) |
| 7 | Limited access to media and information | Limited or no access to the mass media (6)  Low community media saturation (20)  Low community media saturation (21)  Low community media saturation (22)  Low exposure to mass media (35)  Fewer accesses to mass media (44)  Low exposure to mass media (52)  Low exposure to public media (57)  Low media exposure (65)  Low usage of mass media (67)  Low exposure to social media (87)  Low media information (104)  Low use of media (107) |
| 8 | Poor transportation | Distance barrier and poor road network/lack of transportation (7)  Inconvenient transportation (9)  Lack of reliable transportation (10)  Distance to nearest health center (12)  Distance and the dearth of affordable transportation means (17)  Lack of transportation facility (25)  Poor accessibility through road (33)  Limited transportation (35)  Lack of transport means (37)  Transportation barrier (42)  Transportation problems (52)  Poor transportation (56)  Great distance from health facilities (59)  Lack of available transport (60)  Low access (63)  Poor transportation (65)  Poor transportation (70)  Poor transportation (78)  Poor transportation (82)  Poor transportation (88)  Poor transportation (101)  Poor transportation (103)  Transportation problems (117) |
| 9 | Lack of awareness of needs | Pregnancy as anormal life event (9)  Lack of awareness of need (60)  Lack of Importance (97) |
| 10 | Financial restrictions | Non-Affordability of Delivery Charges (7)  Insufficient money for prenatal care (9)  Lack of financial accessibility (15)  Unaffordability (42)  Informal payments (82) |
| 11 | Lack of support from family members | Lack of family support (9)  Lack of women empowerment and support at the household and community (29)  Poor family support (32)  Lack of family consent (36)  Lack of family support (70)  Lack of family support (78)  Lack of family support (82)  Lack of social support (109)  Lack of family support (117) |
| 12 | Lack of spousal support | Lack of husband support (18) |
| 13 | Self-treatment tendencies | Self-assessment of health (11)  Self-treatment and traditional care (94) |
| 14 | Negative attitude towards services | Negative attitude towards preconception care (14)  Having negative attitude on use postnatal services (16)  Resultant concerns about potential side effects (17)  Beliefs about the quality of health services (72)  Dissatisfied with the quality of care (74)  Personal attitude toward care (87) |
| 15 | Cultural and traditional customs barriers | Hindering cultural beliefs and norms (31)  Cultural norms and traditional beliefs (37)  Cultural beliefs (40)  Traditional beliefs (56)  Cultural preference (60)  Cultural beliefs (70)  Traditional beliefs and practices (72)  Dominance of cultural beliefs (73)  Traditional belief (78)  Cultural belief (86)  Cultural belief (88)  Cultural belief (89)  Cultural belief (90)  Cultural and traditional belief (91)  Cultural belief (94)  Cultural belief (99)  Cultural restrictions (103)  Traditional belief (104)  Cultural belief (109)  Traditional beliefs (117) |
| 16 | Negative attitude towards the competence of service providers | Perceived incompetence of providers (40)  The perceived limited midwifery skills (72)  Lack of confidence in providers (72)  Provider’s perceived lack of empathy (74)  Previous bad interactions with health professionals (87)  Fear of Mistreatment by Clinic Staff (96)  Negative attitudes towards male midwives (103)  Previous negative experience with skilled healthcare (109) |
| 17 | Negative experience from past services | Dissatisfaction and previous bad experience (40)  Bad previous experience (56)  Negative Postnatal Care Experiences (96) |
| 18 | Preferences for using traditional services | Using traditional practices (52)  Using traditional services (53)  Experiential judgment of the elderly of the community (72)  Dominance of traditional health workers (73)  Preference for traditional medicine (86)  Preference of traditional medicine (88)  Acceptance of traditional birth attendants (103)  Preference for traditional services (105)  Preference for traditional medicine (115) |
| 19 | Sense of shame and fear | Major source of stigmatization for family planning programs (17)  Fear of experiencing dignity violations (54)  Fear of being ill-treated (54)  Insecurity related fear (54)  Fear of being embarrassed (54)  Perception of lack of privacy (56)  Shyness or embarrassment (77)  Fear of being ill-treated (77) |
| 20 | Communication barriers | Language barrier (94)  Language barrier (109) |

**Challenges in the utilization of Maternal Health Services**

**Thematic Analysis (Categories, sub-themes & themes)**

| **Themes** | **Sub-themes** | **No** | **Categories** |
| --- | --- | --- | --- |
| Knowledge barriers | Weakness of specific knowledge on maternal health | 1 | Lack of awareness of risk factors and danger signs |
|  |  | 2 | Lack of awareness of available maternal health services |
|  |  | 3 | Failure to receive special care and information in previous stages of care |
|  | Weakness of general health knowledge | 7 | Limited access to media and information |
|  |  | 9 | Lack of awareness of needs |
| Barriers related to beliefs, attitudes and preferences | Negative attitude towards the service delivery system | 14 | Negative attitude towards services |
|  |  | 16 | Negative attitude towards the competence of service providers |
|  |  | 17 | Negative experience from past services |
|  | Cultural barriers | 13 | Self-treatment tendencies |
|  |  | 15 | Cultural and traditional customs barriers |
|  |  | 18 | Preferences for using traditional services |
|  |  | 19 | Sense of shame and fear |
|  |  | 20 | Communication barriers |
| Access barriers | Economic barriers | 4 | Poor insurance coverage |
|  |  | 10 | Financial restrictions |
|  | Physical access barriers | 8 | Poor transportation |
| Barriers related to family structure and power | Challenges of autonomy and independence in decision-making | 5 | Limited autonomy and low decision-making power |
|  |  | 6 | Dependence on spouse in decision-making |
|  | Lack of support | 11 | Lack of support from family members |
|  |  | 12 | Lack of spousal support |
